# Hydrophobic Interactions Drive Binding between Vascular Endothelial Growth Factor-A (VEGFA) and Polyphenolic Inhibitors

**DOI:** 10.3390/molecules24152785

**Published:** 2019-07-31

**Authors:** Natalia Perez-Moral, Paul W. Needs, Christina W.A. Moyle, Paul A. Kroon

**Affiliations:** Food Innovation and Health, Quadram Institute Bioscience, Norwich Research Park, Norwich NR4 7UA, UK

**Keywords:** flavonoids, epigallocatechin gallate, tyrosine receptor kinases, angiogenesis, VEGF, protein, polyphenol, surface plasmon resonance (SPR), circular dichroism (CD)

## Abstract

Some polyphenols have been shown to inhibit, at physiological levels, the VEGF-induced VEGF receptor-2 signaling that causes angiogenesis, allegedly by direct interaction with VEGF and reducing the binding to its receptor VEGFR2. Surface plasmon resonance was used to measure the parameters of binding between VEGF and polyphenols as well as the nature of the interactions by assessing the effect of physico-chemical changes in the solution. CD spectrometry was used to determine any change in the secondary structure of the protein upon binding. The kinetic parameters (k_a_, k_d_, and K_D_) that characterise the binding to VEGF were measured for both inhibitor and non-inhibitor polyphenolic molecules. The effect of changes in the physico-chemical conditions of the solution where the binding occurred indicated that the nature of the interactions between VEGF and EGCG was predominantly of a hydrophobic nature. CD studies suggested that a change in the secondary structure of the protein occurred upon binding. Direct interaction and binding between VEGF and polyphenol molecules acting as inhibitors of the signaling of VEGFR2 has been measured for the first time. The binding between VEGF and EGCG seemed to be based on hydrophobic interactions and caused a change in the secondary structure of the protein.

## 1. Introduction

Angiogenesis is a biological process where new blood vessels form from pre-existing ones. Although physiological angiogenesis is a vital and essential process in growth and development, angiogenesis also underpins pathological processes such as the growth of tumours and atherosclerotic plaques. Angiogenesis is regulated by inhibitor and activator molecules that must be balanced to maintain a healthy status, but when the activity of activators like the vascular endothelial growth factor-A (VEGF-A) is excessive, it can act as a powerful angiogenic agent and promote growth of diseased tissues (pathological angiogenesis). 

VEGF-A is an anti-parallel homodimeric glycoprotein. It has been shown to bind to three receptor tyrosine kinases: VEGF receptor 1 (VEGFR1) which has both positive and negative angiogenic effects; VEGFR2 a primary mediator of the mitogenic, angiogenic, and vascular permeability effects of VEGF-A; and VEGFR3, which is involved in the angiogenesis of lymphatic vessels. The VEGF family includes VEGF-A, VEGF-B, VEGF-C, VEGF-D, and the placental growth factor (PLGF). The best studied, VEGF-A, has been very strongly associated with angiogenesis in endothelial cells, and is the target of many anti-angiogenic treatments [1]. The most abundant spliced isoform of VEGF-A is VEGF-A_165_. Throughout this report, VEGF means human VEGFA_165_. 

It has been reported that specific polyphenols found in tea, apples, and cocoa/dark chocolate are potent inhibitors of VEGF-induced VEGF receptor-2 activation, downstream signaling, and angiogenic functions such as vascular endothelial cell proliferation and migration [2]. Although there have been numerous publications presenting evidence that polyphenols can inhibit VEGF signaling, the report of Moyle et al. (2015) was significant in that it provided indirect evidence that inhibition of VEGF signaling caused by specific polyphenols like epigallocatechin gallate (EGCG), found in green tea, and procyanidin oligomers, found in apples and cocoa/dark chocolate, involved binding of the polyphenol to the VEGF protein. In a further report, it was shown that some other polyphenols could also inhibit VEGF signaling by directly interacting with VEGF, while several others did not inhibit VEGF signaling in this way [3]. However, although binding affinities and binding sites on VEGF have been predicted using in silico models [2,3], so far there is no data from experimental studies of binding between VEGF and polyphenols.

Physicochemical data on the kinetics of protein functions (i.e., enzyme activity, ligand binding, transport) and how the protein achieves its functions are fundamental to the understanding and design of strategies to treat related disorders. Blocking certain receptor–ligand interactions can offer a valid approach in drug development, but it is essential to understand the protein–ligand interactions at the molecular level, how they respond to changes in their environment and the mechanisms responsible for the molecular recognition and binding between the protein and ligands. To assess if there is a link between binding and inhibition, we used surface plasmon resonance (SPR) to measure the rate of association and dissociation between different polyphenols that had previously been shown to be potent, moderate, or weak inhibitors of VEGF [2,3]. For the first time, we report evidence of direct binding between polyphenol inhibitors and the VEGF protein and have demonstrated direct interaction between the polyphenols and VEGF protein. We also studied the nature of the interactions between VEGF and EGCG and the effect that binding EGCG has on the secondary structure of the protein by circular dichroism (CD).

## 2. Results and Discussion

### 2.1. Kinetics of Binding between VEGF and Polyphenols

**Figure 1:**
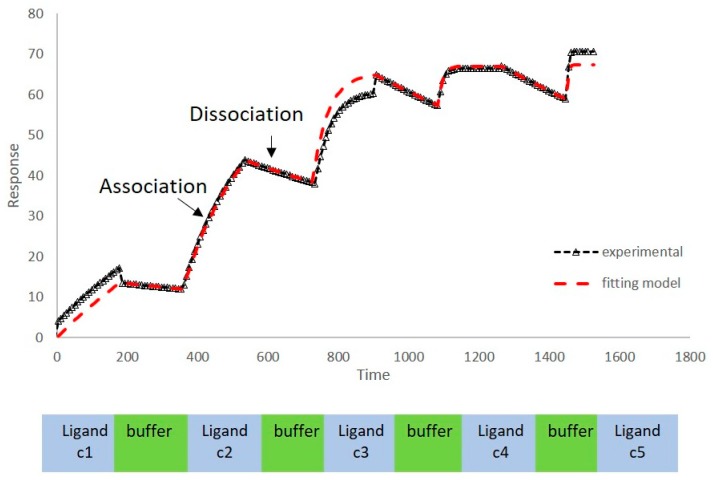
Sensorgram of EGCG binding to VEGF; black triangle—experimental sensorgram; and red line—fitting model. c1–c5 denote ligand concentrations 1 (lowest) to 5 (highest).

We have previously reported a strong correlation between the in silico predicted binding of polyphenols to VEGF and the direct quantification of the inhibition of VEGFR-2 activation caused by exposing VEGF to polyphenols [2,3,4]. However, to date there are no reports providing direct evidence of binding between VEGF and polyphenols, and so we investigated binding interactions using SPR. The SPR sensorgrams showed binding taking place between VEGF and a range of polyphenols in a dose-dependent manner. A representative sensorgram for the binding of the potent VEGF inhibitor EGCG to VEGF is shown in Figure 1. It shows the increase in the response as the binding of EGCG increases with higher concentrations of analytes in a single cycle kinetic assay and the fit with the model used to analyze the results. The estimated kinetic values for the association rate constant (k_a_), dissociation rate constant (k_d_), and equilibrium constant (K_D_) are presented in Table 1, and compared with the concentration of polyphenols required to cause 50% of inhibition of VEGF activation in HUVEC cells (IC_50_) as reported by Cerezo et al. [1]. Structural specificity of polyphenols in their binding to VEGF was apparent from the differences in their kinetic binding constants, with K_D_ values for example ranging from 3.5 × 10^−8^ (epicatechin gallate) to 2.5 × 10^−5^ ((+)-catechin) (Table 1).

The association rate constant k_a_ describes the rate of complex formation, that is the number of VEGF-PP complexes formed per second in a one molar solution of VEGF and PP. Overall, k_a_ values were higher for the more potent VEGF inhibitor polyphenols that exhibited lower IC_50_ values and lower for polyphenols with higher IC_50_, indicating that the more complex formed, the better the inhibition. Likewise, stronger interactions and higher affinity characterised by lower dissociation rate constants (k_d_) and equilibrium dissociation constants (K_D_), correlated generally with lower IC_50_ values that reveal a stronger inhibitory activity. The one exception was myricetin (3′,4′,5′-trihydroxy flavonol) where the kinetic values determined from direct binding experiments by SPR indicated very weak binding, similar to that of (+)-catechin which was a very weak inhibitor of VEGF-dependent VEGFR2 activation (IC_50_ = 215 µM), whereas the IC_50_ for inhibition of VEGF activity by myricetin was 0.121 µM. Myricetin possesses a tri-hydroxy B-ring moiety, and the only other polyphenol with this feature for which we report SPR data is EGCG, which is a very potent inhibitor of VEGF-dependent VEGF activation (IC_50_ = 0.088 µM). However, the potency of EGCG in inhibiting VEGF is most likely due to the presence of the 3-*O*-gallate substitution in the C-ring, because epicatechin-3-*O*-gallate (ECG), which only has a 3′,4′-dihydroxy substituted B-ring, is also a potent inhibitor of VEGF-dependent VEGFR2 activation (IC_50_ = 0.16 µM; Table 1). So how can the relatively potent inhibition of VEGF-dependent VEGFR2 activation by myricetin be explained in the context of it binding only weakly to VEGF? One possibility is that myricetin rapidly (within 5 min) binds to the VEGFR2 that is expressed in vascular endothelial cells, and prevents effective receptor activation by VEGF. Although this was not the case for EGCG and procyanidin dp4 treatment [2], it remains a possibility for myricetin. An alternative explanation is that myricetin is unstable in phosphate buffer and over time is broken down to yield reaction products that do not bind strongly to VEGF, as observed in the SPR measurements. In contrast, in the IC_50_ assays, the myricetin is added directly to a solution of VEGF, allowing myricetin to bind to and inhibit VEGF activity (see [3]). This possibility could be investigated in future studies by assessing possible losses of myricetin and appearance of breakdown products in phosphate buffer over a period of time, and estimating the binding kinetics of any products formed using SPR. 

### 2.2. Effect of Physico-Chemical Conditions on Kinetics of Polyphenol-VEGF Interactions

**Figure 2:**
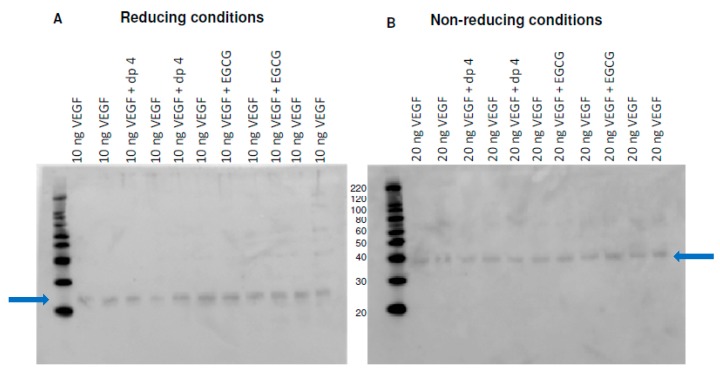
SDS-page gels of control (VEGF) and treatments (VEGF and apple procyanidin fraction dp4 or EGCG under (**A**) reducing conditions and (**B**) Non-reducing conditions.

Binding occurs when intermolecular forces are established between VEGF and the phenolic ligand. SDS page gels strongly suggested that covalent bonds were not responsible for the interaction between VEGF and EGCG, as there was no change in migration of dp4 or EGCG-treated under reducing and non-reducing conditions (see Figure 2). Also, MALDI-TOF MS analyses failed to detect any significant change in the mass of VEGF post-polyphenol treatment as reported in 2015 by Moyle et al. [2]. Therefore, the interactions between VEGF and inhibitor polyphenols are likely to be non-covalent in nature. These forces could be the result of various interactions between the protein, the ligand, and the environment and are likely to be predominantly based on electrostatic (H bonding) and hydrophobic forces [5].

We then studied the nature of the binding interactions between VEGF and polyphenols by examining the effects of changes in temperature, ionic strength, and the presence of chaotropic agents. If the interactions are mainly based on hydrogen bonds some disruptions at higher temperatures or after the addition of a chaotropic agent like urea would be expected. H-bonding interactions would also be challenged by a change in the ionic strength of the environment. Alternatively, if the prevalent binding interactions are based on hydrophobic forces, then a decrease in temperature, and or the polarity of the solvent caused by the incorporation of ethanol, could reduce them.

#### 2.2.1. Effect of Temperature in Binding of VEGF and Polyphenols

**Figure 3:**
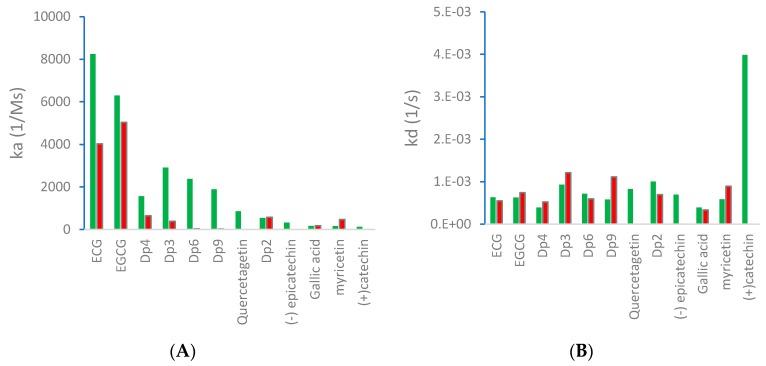
(**A**) Association rate (k_a_) and (**B**) dissociation rate (k_d_) between polyphenols and VEGF at 20 °C (red) and 37 °C (green).

The effect of temperature was studied for several polyphenols. For all the ligands studied except myricetin, the rate of association k_a_ measured by SPR was lower when the binding occurred at 20 °C than at 37 °C, which suggests that hydrophobic interaction was predominant, as hydrophobic forces typically increase with temperature [6]. H-bonding forces, on the other hand, tend to be reduced at higher temperatures. The values for quercetagetin and (+)-catechin could not be determined at 20 °C under the conditions of the analysis, probably because the binding was too weak to be detected by SPR. For EGCG, dp4, dp3, dp9, and myricetin, the dissociation rate was higher at 20 °C than at 37 °C, indicating weaker binding at lower temperatures (Figure 3).

#### 2.2.2. Effect of Environmental Conditions on VEGF-EGCG Interactions at 37 °C 

The addition of a chaotropic agent like urea or salt (NaCl) can disrupt interactions based on H-bonds. However, addition of urea or NaCl to the binding medium did not affect the association rate, and the dissociation rate and K_D_ were reduced indicating stronger interactions. This demonstrated that H-bonding interactions are not the dominant force in the binding of EGCG (Table 2).

Hydrophobic interactions were investigated by varying the polarity of the binding medium. If a change in solution polarity interferes with protein–ligand binding, the interaction is likely to be of a hydrophobic nature. In our system, the incorporation of 10% ethanol in the PBST solution to lower its polarity reduced the VEGF to EGCG association, consistent with hydrophobic interactions. This reduction was even more pronounced in a PBST solution with 30% ethanol. The dissociation rate was increased when 10% ethanol was added in the system, but when the presence of ethanol was increased to 30%, the dissociation was reduced, possibly due to the low number of interactions established initially (Table 2). 

The addition of ethylene glycol to the binding medium had two distinct effects. It decreased the polarity of the environment but also markedly increased the viscosity of the solvent; this could slow the rate of diffusion of the ligand to the binding site. The combination of these two effects, and particularly the increase in viscosity and the subsequent limitation in mass transfer, would explain the important reduction observed in the k_a_ and k_d_, as well as the higher K_D_ measured when compared to binding performed in just PBST. 

Overall, we have shown that hydrophobic forces are the main drivers of initial interactions between the polyphenols and the VEGF protein.

### 2.3. Circular Dichroism

**Figure 4:**
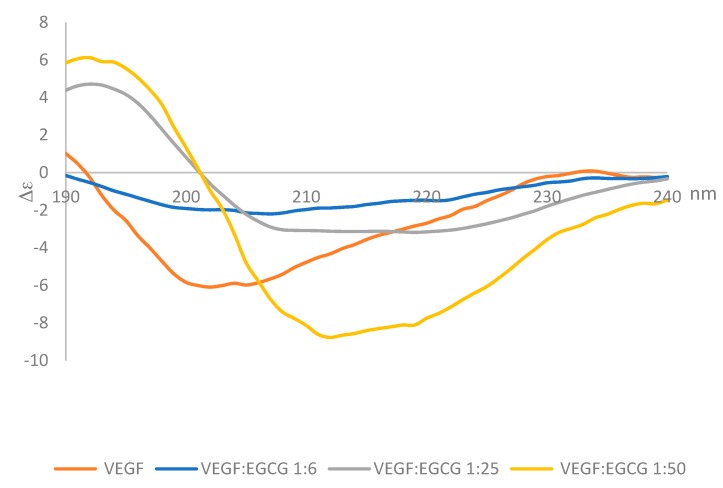
CD spectra of VEGF (blue) and at 3 min after mixing VEGF:EGCG at a 1:6; 1:25, and 1:50 molar ratio using 25 µL of VEGF 0.2 mg·mL^−1^ and 25 µL of EGCG 0, 0.025; 0.1 and 0.2 mg·mL^−1^.

Circular dichroism (CD) in the far UV range (180–260 nm) was used to determine whether the secondary structure of VEGF changed upon binding EGCG. Circular dichroism spectra were measured for a fixed concentration of VEGF-165 (0.22 nmoles in 50 µL) with increasing amounts of EGCG (0, 1.36, 5.45 and 10.91 nmoles) and the spectra obtained are shown in Figure 4. Analysis of the spectra indicated the predominant features in the secondary structure of VEGF165 were random coil (unordered) and β-sheet, followed by smaller percentages of α-helix and turns. After adding EGCG at a mole ratio of 1:50 VEGF:EGCG, the VEGF secondary structure was altered with increases of 65% and 33% in α-helix and turns respectively, a reduction in the percentage of β-strands of 88%, and only a 3% reduction in the predominant unordered structure.

**Figure 5:**
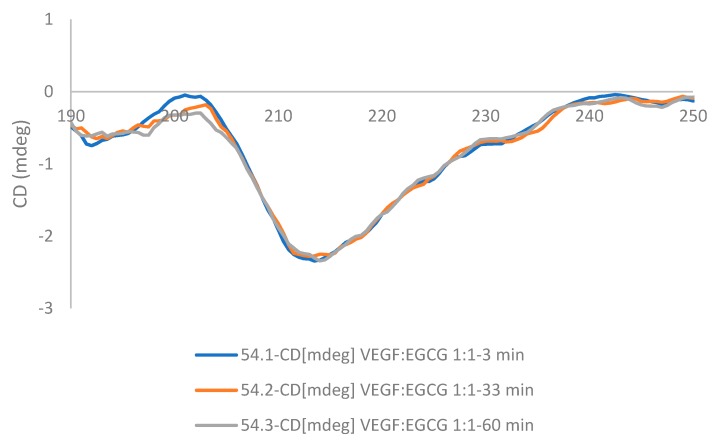
CD spectra of VEGF:EGCG 1:1 after mixing at 3 min (blue line), 30 min (orange line) and 60 min (grey line).

In the CD far UV spectra there was a shift in the position of the negative band from 201 nm for VEGF alone in solution to 211 nm when EGCG was mixed with the VEGF protein. The bands also became more intense as the VEGF:EGCG ratio decreased indicating changes in the conformation of the protein due to the interactions with the EGCG. The spectra obtained 3 min after mixing the protein with the EGCG and 30 min after mixing were identical, suggesting that the structural changes caused by binding occurred rapidly and they were not followed by additional changes (Figure 5). 

**Figure 6:**
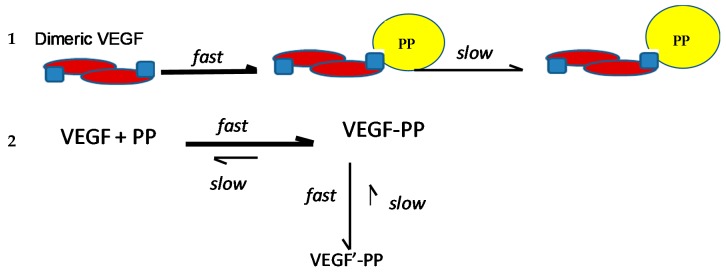
Possible models for binding between VEGF and polyphenols (PP).

Moyle et al. [2] reported a two-phased kinetics for the inhibition of VEGF by EGCG and suggested two explanations. One possibility was that the initial binding of EGCG to VEGF induced relatively rapid but partial inhibition of VEGF activity; which was followed by a conformational change in VEGF that was associated with a further inhibition of its activity. The data presented here show that the conformational changes in VEGF are rapid, taking less than 90 s, whereas the inhibition kinetics occurred over minutes to hours. However, the inhibition kinetics were done with an EGCG concentration of 62.5 nM, orders of magnitude lower than used in the CD studies, so loss of activity as a consequence of the conformational changes is a possibility. An alternative explanation for the biphasic inhibition kinetics reported by Moyle et al. relates to the homodimeric nature of active VEGF, which means there are two identical binding sites for polyphenol inhibitors within each dimer of VEGF. It is possible that the binding of a first polyphenol ligand to one of the two binding sites in a VEGF homodimer is relatively rapid (and driven in part by there being two binding sites per dimer), which partially inhibits its VEGFR2 activation activity, while the binding of a second polyphenol molecule which is required for complete inhibition of VEGF activity occurs more slowly (Figure 6). The binding of the second polyphenol molecule to VEGF would be expected to be slower because the number of available binding sites per VEGF homodimer is one half of that in the unbound state and may also be a consequence of conformational changes in VEGF dimers that occur rapidly upon binding, as shown here using CD.

## 3. Materials and Methods 

### 3.1. Materials

Human Recombinant VEGF165 was purchased from ReliaTech GmbH (Wolfenbüttel, Germany). SPR measurements were performed on a Biacore X100 with carboxymethylated dextran CM5 sensor chips (GE Healthcare Life Sciences, Little Chalfont, Buckinghamshire, UK). Epicatechin gallate (ECG), quercetagenin, (−)-epicatechin, and epigallocatechin were purchased from Extrasynthese (Lyon, France). Epigallocatechin gallate (EGCG) was obtained from Toronto Research Chemicals Inc. Gallic acid, myricetin, (+)-catechin, *N*-hydroxysuccinimide (NHS), *N*-ethyl-*N*′-(3-dimethylaminopropyl) carbodiimide hydrochloride (EDC), ethanolamine, phosphate buffered saline (PBS), sodium acetate, tween 20, glycine, and any other reagent were from Sigma. A series of purified procyanidin fractions with different degrees of polymerisation (dp2, dp3, dp4, dp4, dp6, and dp9) were prepared in the lab following a previously described procedure [7].

### 3.2. Surface Plasmon Resonance Studies

VEGF165 was covalently immobilised to the surface of a CM5 chip following a modification of the protocol described in Chen et al. [8] by treating the chip with 400 mM EDC and 100 mM NHS before immobilising VEGF165 dissolved in 20 mM sodium acetate at pH 4.8 (11.9 µg/mL) until it reached a response of 3120 RU. The untreated reactive succinimide groups were then quenched with 1 M ethanolamine at pH 8.5. The immobilisation of VEGF was validated subsequently with heparin. In the binding studies different polyphenols (PP) at several concentrations varying between 0 and 25 µg/mL were solubilised in PBS buffer containing 0.05% tween 20 and 1% DMSO (PBST) and were injected at a flow rate of 30 µL/min with a contact time of 180 s and 600 s of dissociation time either at 20 or 37 °C. Polyphenol molecules were initially dissolved in DMSO to help solubilising them before being diluted in PBST to a final concentration of 1% DMSO. The binding interactions were measured in PBST with 1% DMSO as running buffer, or in PBST 1%DMSO with the environmental modifiers (urea, NaCl, ethanol, or ethylene glycol). Different polyphenols expected to behave as inhibitors or non-inhibitors for the stimulation of VEGF receptor 2 (VEGFR-2) were examined [3]. The data analyses were performed with experimental data points fitting a homogeneous 1:1 Langmuir binding model as a valid method for kinetic ranking of different molecules binding to VEGF (Figure 1).

All the sensorgrams were double referenced before proceeding to evaluation by subtracting the non-specific binding to a reference cell with no VEGF bound and the values obtained after injecting a blank buffer. Solvent effects caused by the presence of DMSO were also corrected. Sensor chips were regenerated by treating them with two pulses of 10 mM glycine at pH 2.

### 3.3. Far-UV Circular Dichroism Spectroscopy

Circular Dichroism (CD) spectra were recorded using a Jasco J-715 spectropolarimeter (Easton, MD, USA) over a wavelength range of 185–260 nm in PBS solvent with 1% ethanol, using a quartz demountable CD cell with a path length of 0.1 mm. Averages of three runs of the protein spectra were collected a 37 °C at a scan speed of 20 nm/min and a band width of 1.0 nm. To account for any background effect the protein in buffer alone, the EGCG molecule in buffer alone, and the buffer alone were also measured. After background correction the spectra were converted to units of delta epsilon. Dichroweb analysis server http://dichroweb.cryst.bbk.ac.uk [9,10] was used to analyse and estimate on line the secondary structure contents.

## 4. Conclusions

Data presented here has shown that some but not all polyphenols can bind to VEGF and that there is a strong correlation between the strength of binding interactions and the potency of the polyphenol inhibitor. Hydrophobic forces appeared to be the main supramolecular forces driving the interaction between EGCG and VEGF. A study of the secondary structure of the protein showed that VEGF undergoes conformational changes upon binding to EGCG, that these changes occurred during the first minutes of interaction and that the effect was increased with larger amounts of PP. Overall, these findings are significant in confirming VEGF as a molecular target for polyphenols that are prevalent in human diets.

## Figures and Tables

**Table 1:** Kinetic values of the association rate constant k_a_, dissociation rate constant k_d_, equilibrium constant K_D_ in PBST at 37 °C and IC_50_ [1] for different polyphenols

	k_a_ (1/Ms)	k_d_ (1/s)	K_D_ (M)	IC_50_ (µM)	
Epicatechin gallate (ECG)	8240	6.33 × 10^−4^	3.48 × 10^−8^	0.16	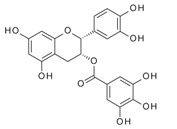
Epigallocatechin gallate (EGCG)	6300	6.25 × 10^−4^	1.12 × 10^−7^	0.09	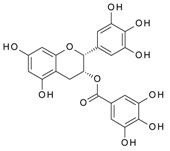
Procyanidin Dp3	2910	9.30 × 10^−4^	3.51 × 10^−7^	0.78	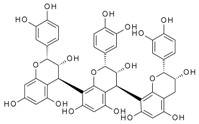
Procyanidin Dp6	2377	7.14 × 10^−4^	9.79 × 10^−6^	ND	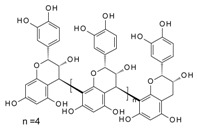
Procyanidin Dp9	1893.5	5.81 × 10^−4^	3.41 × 10^−7^	ND	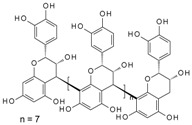
Procyanidin Dp4	1566.5	3.91 × 10^−4^	2.77 × 10^−7^	0.28	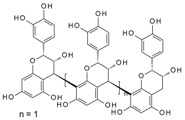
Quercetagetin	857.7	8.26 × 10^−4^	9.34 × 10^−7^	0.10	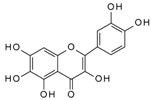
Procyanidin Dp2	548.4	1.00 × 10^−3^	6.37 × 10^−7^	52.6	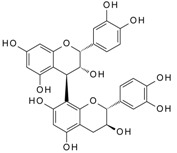
(-) Epicatechin	323.1	6.96 × 10^-4^	2.71 × 10^-6^	ND	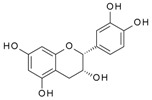
Gallic acid	166.3	3.91 × 10^−4^	5.52 × 10^−6^	ND	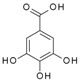
Myricetin	153.4	5.83 × 10^−4^	4.46 × 10^−6^	0.12	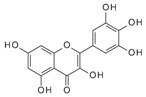
(+) Catechin	130.6	3.99 × 10^−3^	2.46 × 10^−5^	215	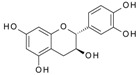

ND: not determined.

**Table 2:** Binding parameters (ka, kd, and KD) between EGCG and VEGF-165 under different physico-chemical environments

	ka (1/Ms)	kd (1/s)	KD (M)
PBST	5184 ± 64	8.8 × 10^−4^ ± 5.2 × 10^−5^	1.7 × 10^−7^
PBST + 1 M urea	5160 ± 100	3.6 × 10^−4^ ± 2.1 × 10^−5^	7.1 × 10^−8^
PBST	6147 ± 52	6.8 × 10^−4^ ± 2.9 × 10^−6^	1.1 × 10^−7^
PBST + 0.5 M NaCl	5184.3 ± 92	3.7 × 10^−4^ ± 4 × 10^−5^	6.0 × 10^−8^
PBST + 1 M NaCl	6502.2 ± 260	3.3 × 10^−4^ ± 4 × 10^−5^	3.5 × 10^−8^
PBST	6052 ± 64	7.2 × 10^−4^ ± 5 × 10^−5^	1.4 × 10^−7^
PBST + 10% ethanol	2464.7 ± 60	1.7 × 10^−3^ ± 7 × 10^−5^	8.1 × 10^−7^
PBST + 30% ethanol	481.9 ± 21	2.0 × 10^−4^ ± 5 × 10^−5^	4.7 × 10^−7^
PBST	6147 ± 52	6.8 × 10^−4^ ± 2.9 × 10^−6^	1.1 × 10^−7^
PBST + 10 M ethylene glycol	796.1 ± 45	2.6 × 10^−4^ ± 5.2 × 10^−5^	4.2 × 10^−7^
